# Ethical Conflict and Its Psychological Correlates among Hospital Nurses in the Pandemic: A Cross-Sectional Study within Swiss COVID-19 and Non-COVID-19 Wards

**DOI:** 10.3390/ijerph182212012

**Published:** 2021-11-16

**Authors:** Michele Villa, Colette Balice-Bourgois, Angela Tolotti, Anna Falcó-Pegueroles, Serena Barello, Elena Corina Luca, Luca Clivio, Annette Biegger, Dario Valcarenghi, Loris Bonetti

**Affiliations:** 1 Cardiocentro Ticino Institute, Ente Ospedaliero Cantonale (EOC), Via Tesserete 48, 6900 Lugano, Switzerland; michele.villa@eoc.ch; 2 Pediatric Institute of Southern Switzerland, Ente Ospedaliero Cantonale (EOC), Via Gallino, 12, 6500 Bellinzona, Switzerland; colette.balice@eoc.ch; 3 Nursing Development and Research Unit, Oncology Institute of Southern Switzerland, Ente Ospedaliero Cantonale (EOC), Via Gallino, 12, 6500 Bellinzona, Switzerland; dario.valcarenghi@libero.it; 4 School of Nursing, Faculty of Medicine and Health Sciences, Consolidated Research Group SGR 269 Quantitative Psychology, University of Barcelona (Spain), Campus Bellvitge, Pavelló de Govern, 3a planta, Despatx 331, Feixa Llarga, s/n L’Hospitalet de Llobregat, 08907 Barcelona, Spain; annafalco@ub.edu; 5 EngageMinds HUB—Consumer, Food & Health Engagement Research Center, Department of Phychology, Università Cattolica del Sacro Cuore, Milano and Cremona, L.Go Gemelli 1, 20123 Milan, Italy; serena.barello@unicatt.it; 6 Ospedale Regionale di Lugano, Ente Ospedaliero Cantonale (EOC), Via Tesserete, 46, 6903 Lugano, Switzerland; Corinaelena.luca@eoc.ch; 7 ICT Data Science & Research Unit, Ente Ospedaliero Cantonale (EOC), 6500 Bellinzona, Switzerland; Luca.clivio@eoc.ch; 8 Nursing Department Direction, Ente Ospedaliero Cantonale (EOC), Viale Officina, 3, 6500 Bellinzonal, Switzerland; annette.biegger@eoc.ch; 9 Nursing Research Competence Centre, Nursing Direction Department, Ente Ospedaliero Cantonale (EOC), Viale Officina, 3, 6500 Bellinzona, Switzerland; loris.bonetti@eoc.ch; 10 Department of Business Economics, University of Applied Sciences and Arts of Southern Switzerland, Health and Social Care, Via Violino, 11, 6928 Manno, Switzerland

**Keywords:** ethical conflict, nurse, resilience, psychological distress, SARS-Cov-2, Covid-19, pandemic

## Abstract

Background: During the Covid-19 pandemic, nurses experienced increased pressure. Consequently, ethical concerns and psychological distress emerged. This study aimed to assess nurses’ ethical conflict, resilience and psychological impact, and compare these variables between nurses who worked in Covid-19 wards and nurses who did not. Methods: Design—Multicentre online survey. Setting—Multi-site public hospital; all nursing staff were invited to participate. The survey included validated tools and a novel instrument to assess ethical conflict. Spearman’s rho coefficient was used to assess correlations between ethical conflict and psychological distress, logistic regressions to evaluate relationships between nurses’ characteristics and outcome variables, and the Mann–Whitney/t-test to compare groups. Results: 548 questionnaires out of 2039 were returned (275 = Covid-19; 273 = non-Covid-19). We found a low–moderate level of ethical conflict (median = 111.5 [76–152]), which emerged mostly for seeing patients dying alone. A moderate and significant positive correlation emerged between ethical conflict and psychological distress *rs* (546) = 0.453, *p* < 0.001. Nurses working in Covid-19-ICUs (OR = 7.18; 95%CI = 3.96–13.01; *p* < 0.001) and Covid-19 wards (OR = 5.85; 95%CI = 3.56–9.6; *p* < 0.001) showed higher ethical conflict. Resilience was a protective factor for ethical conflict. Conclusions: Ethical conflict was significantly linked to psychological distress, while a higher level of resilience was found to be a protective factor. These results can be informative for nursing management in future similar crises.

## 1. Introduction

The Covid-19 pandemic raises major public health concerns around the world. This new disease, which began to spread in China’s Wuhan region in late 2019, is associated with high morbidity and mortality rates [1]. Across the world, Covid-19 has already affected a high percentage of the population: at the time of writing, the epidemiological situation worldwide is still critical, with a total of 175,333,154 confirmed cases and 3,793,230 deaths. Switzerland and the Principality of Liechtenstein have reported 701,260 cases and 10,315 deaths (WHO Covid-19 Dashboard, 16 June 2021).

On a physical and psychological level, healthcare workers in hospitals, and nurses in particular, who are in constant contact with patients, constitute one of the groups at higher risk [2]. In fact, the literature has recently demonstrated that the Covid-19 pandemic—similarly to previous ones—has had many consequences on nurses, who experienced great fatigue for several reasons: direct exposure to infected or dying patients, insufficient personal protective equipment, and the exposure to the risk of contamination for themselves and their families [2,3,4,5,6,7,8]. High levels of psychological distress, anxiety and depression also emerged [8,9,10], with short-term and long-term consequences, such as insomnia, burnout and post-traumatic stress symptoms [6]. Moreover, research found that psychological resilience often constituted a potentially protective factor for the development of psychological sequelae [11].

Furthermore, this extremely challenging situation has required nurses to cope with complex clinical decisions, thus exposing them to frequent ethical conflicts [3,4,5,6,12,13,14,15,16,17]. Ethical conflict is defined as a problem that arises when the idea of “good” or “right” with regard to other people’s welfare or best interests or self-interest is compromised [18]. It is a complex construct that involves not only the inability to perform and ethical action due to the existence of barriers, as well as moral distress, but also other problematic situations such as the difficulty in identifying conflicting values or principles, moral uncertainty, having difficulty in choosing between two moral values, moral dilemmas, or being witness to someone violating the ethical principles assumed by oneself [19,20,21]. In the same line, ethical conflicts can represent a difficulty in the decision-making process, which affects the healthcare professional [22].

Various authors have stated that, during the Covid-19 pandemic, the ethical conflicts experienced by nurses—which were more intense in the critical care units—stemmed from three main sources, namely: (i) the relationship with patients and their families, which required a total revision of communication techniques and schemes; (ii) the provision of several treatments; and (iii) the specific characteristics of the setting in which the clinical team was asked to work. With regard to the first source, the decision-making process clashed with issues such as the difficulty of ensuring adequate informed consent to the patients, a failure to respect confidentiality, and a lack of protection of the patients’ interests [13,14,15,16,17,23]. With respect to the provision of treatments, the Covid-19 pandemic forced nurses to experience conflict when asked to administer treatments they perceived as overly aggressive, when pain management seemed to be lacking, or when it became necessary to limit the use of life support procedures, especially in relation to End-of-Life care [13,14,15,16,17,23]. Finally, in relation to workplace dynamics, ethical conflict frequently emerged when professionals working in extreme conditions were not fully involved in the decision-making process or when the work environment made it difficult to consider issues of a bioethical nature [13,14,15,16,17,23]. Thus, it is clear that the Covid-19 pandemic frequently exposed nurses to these situations, which have the potential to produce different ethical conflicts in each person. These conflicts may ultimately constitute a relevant risk factor for the development of psychological issues, in both the short- and the long-term.

## 2. Aims of the Study

This study aimed to assess the impact of the Covid-19 pandemic on hospital nurses in the Italian-speaking region of Switzerland in terms of exposure to ethical conflict and psychological distress. More specifically, the objectives of this study were twofold: (1) to analyse whether ethical conflict is associated with psychological distress and whether this relationship is conditional on individual levels of resilience and work-related characteristics among nurses working during the Covid-19 pandemic; and (2) to compare the experiences of nurses who worked in Covid-19 wards and those who worked in non-Covid wards. For the purpose of the study, it is hypothesised that Covid-19-related ethical conflict is associated with the risk of psychological distress and that higher levels of resilience can be protective in terms of both ethical conflict and psychological distress.

## 3. Materials and Methods

### 3.1. Study Design

A multicentre, cross-sectional, observational study was conducted among hospital nurses working at the Ente Ospedaliero Cantonale, a multi-site public hospital—comprising eight hospitals in different locations—in the Italian-speaking region of Switzerland.

### 3.2. Setting and Sample

With more than 5000 employees, the Ente Ospedaliero Cantonale, located in the Italian-speaking region of Switzerland, provides services to a population of 350,000 people and the eight hospital sites are geographically distributed in order to be reachable from all areas of the canton. Seven hospitals out of eight are general hospitals covering all medical specialties, and one is a rehabilitation institute.

During the first and second wave of the Covid-19 pandemic, the public hospital changed its organisation and dedicated two of its main sites to the care of patients with Covid-19. To cope with the increasing number of patients, the number of beds in the intensive care units (ICU) was also increased, along with the creation of new ICUs. In this new context, many nurses were asked to work in different care units from their usually assigned ones. In some cases, they were asked to work in ICUs without previous specific training or sufficient knowledge of the critical care setting. Despite the professional quality of the nurses and their willingness to attend to the growing volume of patients as adequately as possible, this situation created several difficulties, leaving the staff under pressure and with a higher workload.

All nurses (in total 2039) working at the public hospital were invited to participate in the research. The decision was made to involve all employed nurses because, although not all of them were directly involved in the care of Covid-19 patients, from an organizational point of view, the clinical setting of the hospital was completely reshaped during the pandemic. In fact, the care activities changed also in non-Covid-19 units because many nurses were moved to Covid-19 units and those who remained in their assigned unit had to deal with a heavier workload compared to the pre-Covid period. Therefore, each nurse was, in some way, influenced professionally and personally by the pandemic.

Participation in the survey was voluntary. The participants provided their informed consent via the specially created survey platform.

### 3.3. Questionnaires and Data Collection

Three questionnaires and an ad-hoc form were used to collect data through an online, self-administered survey. The software was created by the institutional Information Technology Department. In total, the questionnaire had 65 items and 16 screens. The nurses were invited to complete the survey using their smartphones or computers via email at their institutional mailboxes. Data collection started in July 2020 (after the first wave of the pandemic) and ended in September 2020. To increase the response rate, two reminders were sent by email to the nurses, following the invitation to participate in the survey. To incentivise survey participation, the nurses could complete the survey during their work time. The system avoided missing values by forcing the participants to answer to the missing items before switching to a new page. The respondents were able to review and change their answers through a back button.

#### 3.3.1. Socio-Demographic and Work-Related Characteristics

The questionnaire included socio-demographic topics (i.e., gender, age, marital status, if the nurse lived alone or with vulnerable people (older people or children), level of education, and perceived health status) and work-related topics (i.e., length of work experience; hospital department during the pandemic; if the nurse changed department during the pandemic; if the nurse worked in a Covid-19 ward during the pandemic and, if so, when he/she started and for how long; the desire to change profession before and during the pandemic; self-perceived general risk; and self-perceived risk of infection).

#### 3.3.2. Validation of the Ethical Conflict Scale Covid-19 (ECS-Co19)

To assess the nurses’ moral challenges in terms of ethical conflict during the Covid-19 pandemic, the authors developed a new tool, namely the *Ethical Conflict Scale Covid-19 (ECS-Co19)*. A systematic approach was followed for the creation of the ECS-Co19, which was developed by four authors (MV, AFP, SR, and LB). Firstly, a member of the research team (MV) interviewed the nurses who worked with Covid-19 patients to understand which ethical issues they had been experiencing during the pandemic. Secondly, two authors (LB and AFP)*,* who are specialists in ethical dimensions of care, developed the items of the scale, based on the nurses’ interviews and the available literature on ethical conflict during Covid-19 [14,15,24,25,26]. These items were then organized into five main categories [14]: (1) Resources (i.e., the availability of material and human resources for addressing the requested care needs); (2) Protection (i.e., the perceived adequate protection for healthcare workers to prevent a Covid-19 infection); (3) Decisions (i.e., the decision-making process); (4) End-of-Life care and withholding and withdrawal of treatment (i.e., the management of End-of-Life and treatment); (5) Information and communication technologies (i.e., the use of Information Communication Technology (ICT) to maintain contact with relatives during the confinement and ward access restrictions). In addition, SB, an expert psychologist in psychometric tool development and testing, evaluated the items for their content and semantic structure. Once completed, the ECS-Co19 was pilot tested with 10 nurses for content and face validity. In order to assess the stability of the tool, the same nurses were also involved in the test–retest procedure.

The ECS-Co19 consisted of 19 preliminary statements about possible situations of ethical conflict, answered in terms of the frequency (from never = 1 to very often = 5) and intensity of the perceived ethical conflict (from none = 1 to very high = 5). Similarly to other tools that measure ethical conflict [27], the ECS-Co19’s score is the sum of the product of frequency and intensity of all the items. The higher the score, the higher the level of ethical conflict. To assess its reliability, Cronbach’s alpha was used for item consistency, the Intraclass Correlation Coefficient (ICC), measuring absolute agreement, was used to assess the stability of the tool, and the Exploratory Factorial Analysis (EFA) was used to assess construct validity. The Kaiser–Meyer–Olkin (KMO) test of sample adequacy and the Bartlett’s test of sphericity were conducted prior to factor extraction. The factors were extracted by means of the principal axis factorial extraction. A parallel analysis and a scree plot were used to identify the number of factors to be extracted [28,29]. Loading values of >0.30 were considered valid [30].

The results of the pilot test showed a good level of content and face validity: the nurses found the tool simple and consistent with their experience of ethical issues during the pandemic: they suggested minor changes in two items to improve the readability of the survey and the deletion of one item that was not consistent with the situation. The final version of ECS-Co19 is reported in Supplementary Materials S1.

#### 3.3.3. Self-Reported Psychological Distress

To assess psychological distress, the Italian version of the Impact of Event Scale—Revised (IES-R) [31] was used, which measures individual stress reactions after traumatic events. The Italian version of the IES-R has 22 items, which can be answered with a 5-point Likert scale ranging from Not at all = 0 to Extremely = 4. The IES-R has three subscales: intrusion (α = 0.78); avoidance (α = 0.72); and hyper-arousal (α = 0.83). The IES-R score ranges from 0 to 0.88. A score of over 30 is considered a cut-off for post traumatic syndrome symptomatology.

#### 3.3.4. Psychological Resilience

Resilience was measured using the Brief Resilience Scale (BRS) [32]. The BRS, which aims to evaluate the ability of an individual to recover from stressful events, encompasses six items. The BRS has a 5-point Likert response (from 1 = strongly disagree, to 5 = strongly agree): given the one-factor structure of the scale, the higher the score, the greater the resilience. The BRS is mono dimensional, with a Cronbach’s alpha(α) ranging from 0.80 to 0.91 and an ICC ranging from 0.61 to.69 [32]. Items 2, 4, and 6 have a reverse coding.

## 4. Data Analysis

Data were analysed using SPSS Version 26 (IBM Corp., New York, NY, USA). Statistical significance was assumed for *p* < 0.05. Descriptive statistics were implemented to determine the distribution of sociodemographic characteristics, including gender, age, education level, hospital department, etc. Categorical variables were expressed as absolute values and percentages. Continuous variables were reported as means and standard deviations or median [25th, 75th percentiles], where appropriate.

The differential distribution of the characteristics assessed by the survey in relation to the experience of nurses in Covid-19 wards was analysed using the Chi-square test or Fisher’s exact test for categorical variables. Continuous variables were compared using the unpaired t test or the non-parametric Mann–Whitney U test. A comparison of each item of the ethical conflict scale was made between nurses who worked in Covid-19 wards and those who worked in non-Covid-19 wards.

In addition, a comparison of the total score and the three IES-R subscales was made between nurses who worked in Covid-19 wards and those who worked in non-Covid-19 wards, by means of a Mann–Whitney U test. The nurses’ level of resilience was calculated as the median value of the BRS score [32], and compared between those who worked in Covid-19 wards and those who did not with a Mann–Whitney U test. Spearman’s correlation was applied to assess the correlation between ethical conflict, psychological distress, and resilience.

To better understand the relationship between ethical conflict and the other variables, the overall score of the ethical conflict scale was divided into quartiles (Q1, Q2, and Q3) and dichotomised as follows: “Low Ethical Conflict” = Q1 plus Q2; “High Ethical Conflict” = Q3. In addition, the IES-R was dichotomised considering the cut-off point reported in the literature, namely >30 points as the cut-off for an initial post-traumatic stress syndrome symptomatology [31].

Finally, a multivariate logistic regression analysis was performed in order to identify characteristics associated with “High Ethical Conflict” and IES-R > 30 points. The model included the main variables potentially associated with ethical conflict and psychological distress, namely gender, professional experience, level of education, level of resilience, desire to change job before and during the pandemic, full-time employment, and type of hospital department worked in during the pandemic (non-Covid-19 ward, Covid-19 ward, non-Covid-19 ICU, and Covid-19 ICU).

The Hosmer–Lemeshow test was used to assess the calibration of the model. The predictive ability of the model to discriminate between nurses who experienced any consequences and those who did not was estimated using Receiver Operating Characteristic (ROC) curves (Supplementary Materials S2). The odds ratio (OR) and corresponding 95% confidence intervals (95% CI) were calculated for each independent variable.

## 5. Results

### 5.1. Socio-Demographic Characteristics

A total of 548 nurses out of the 2039 invited to participate in the study returned the questionnaires completed, thus representing a response rate of 27.4%. The participants were mostly female (76.5%), with a mean age of 40 ± 10 years. The majority had more than 20 years of work experience (39.2%), achieved a post-bachelor-degree education (57.8%), lived with frail people (46.9%), and had a work-time percentage higher than 90% (54.9%). Of these nurses, 50.2% worked in a Covid-19 unit during the first pandemic wave, while 30.5% were relocated to a different unit from the one where they usually worked. The participants perceived their health status as good (median = 4 [2–4]) and stated a moderate level of perceived risk of infection by Covid-19 (median = 5 [4–8]). Interestingly, the desire to change profession decreased during the pandemic (21.7% during the pandemic versus 26.8% before the pandemic).

The main characteristics of the participants, also considering their work settings (i.e., Covid-19 Unit vs. non-Covid-19 Unit), are reported in Table 1.

### 5.2. Validity and Reliability of the Ethical Conflict Scale Covid-19 (ECS-Co19)

The sample met the criteria for EFA (KMO = 0.90, Bartlett *p*-value < 0.001). One item was deleted due to having a factor loading lower than 0.30. The final scale (17 items) revealed a one-factor solution accounting for 33.5% of variance, explaining the main ethical dimension of the questionnaire. Loading factors ranged from 0.38 to 0.73. The overall Cronbach’s alpha was 0.89 and did not change if any item was removed. The ICC was good–very good, ranging from 0.75 to 0.99 (Supplementary Material S1). The final version of the tool, with 17 items, has a score between 17 and 425.

### 5.3. Ethical Conflict

**Figure 1:**
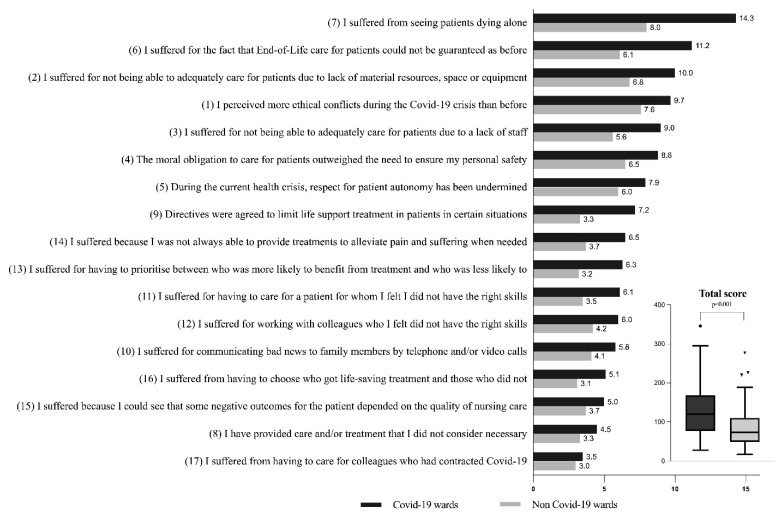
Ethical conflict items, comparison between Covid-19 and non-Covid-19 wards (values are shown as means).

The total sample showed a median level of ethical conflict of 111.5 [76–152]. This was significantly different (*p* < 0.001) when comparing Covid-19-unit nurses (median = 133 [91–179]) to non-Covid-19-unit nurses (median= 91 [68.5–125]) (Table 2, Figure 1), in the following areas: “Resources, Protection, End-of-Life care, Information and Communication Technologies and Decision making process”. Moreover, the single items resulted in significant differences between the two groups, with the exception of item 17 “I suffered from having to care for colleagues who had contracted Covid-19” (*p* = 0.988). The items with the highest score were “I suffered from seeing patients dying alone” and “I suffered from the fact that End-of-Life care for patients could not be guaranteed as before” (Table 2).

#### Psychological Distress

Overall, the nurses in our sample showed a moderate level of psychological distress (median = 15 [7–26]).

However, a total of 109 nurses (19.9%) reported a score greater than 30 on the IES-R score, which is considered a cut-off for initial post-traumatic stress syndrome symptomatology. In this regard, there was a significant difference between Covid-19 and non-Covid-19 wards, with a higher percentage in the former.

Comparing the overall score of perceived psychological stress between those who worked with Covid-19 patients and those who did not, there were greater stress levels among the first group, although, in both populations, the level remained low, with a median score of 17 [7–29] among those who worked in Covid-19 wards and 12 [6–23.5] among those who did not (Table 3).

### 5.4. Resilience

With regard to the level of resilience, participants showed a medium-high score (Table 4), i.e., a good level of resilience, considering both their resilience in a normal situation, as well as the resilience shown during the pandemic, with a median score of 3 for both. With respect to this variable, no significant difference was found between Covid-19 and non-Covid-19 units.

### 5.5. Risk Factors Associated with High Ethical Conflict and High Psychological Distress

Spearman’s test estimated a statistically moderate significant positive correlation between the Ethical Conflict score and the IES-R, *rs* (546) = 0.453, *p* < 0.001.

With regard to the predictive variables for ethical conflict, the logistic regression model was statistically significant, χ2(9) = 101.594, *p* < 0.001, explaining 23.5% (Nagelkerke R2) of the variance.

The likelihood of experiencing higher levels of ethical conflict increased if the nurse was female and worked in Covid-19 wards and Covid-19-ICUs. In addition, working in general ICUs increased the probability of experiencing higher levels of ethical conflict. In particular, there was an increased likelihood of female nurses working solely in Covid-19 wards (i.e., not ICUs) experiencing symptoms of psychological distress. On the other hand, higher levels of resilience and more years of working experience were found to be protective factors against ethical conflict (Table 5).

## 6. Discussion

This study aimed to evaluate the moral challenges of nurses in terms of ethical conflict during the Covid-19 pandemic and its impact on psychological distress. In addition, potential protective factors against ethical conflict and psychological distress were also investigated.

The sudden outbreak of the pandemic showed the vulnerability of human health and the response capacity and limits of health resources in facing a serious health situation on a global scale. The well-documented impact of the pandemic on nurses and nursing, particularly in critical care settings, ranges from depression, anxiety, emotional withdrawal, frustration, and anger to a variety of physical symptoms. Despite the vast literature available, only a few studies have analysed ethical conflict and psychological distress in depth, and how resilience may play a protective role in both these phenomena.

The results of this survey outlined a low–moderate level of ethical conflict in the general sample, but there were significant differences in the total score and, in the majority of the items, between nurses who worked in Covid-19 units and those who did not. Considering the median scores, Covid-19-unit nurses experienced 68% more ethical conflict than non-Covid-19 nurses. These results are in keeping with other recent studies [6,14,33,34]. Furthermore, the survey revealed that nurses from both populations perceived an increased level of ethical conflict during the pandemic compared to the past. Issues related to End-of-Life care were considered an important cause of ethical conflict in Covid-19-unit nurses, thus confirming evidence from previous research [14,15,18,35]. Not guaranteeing the care that nurses consider satisfactory or not being able to accompany the patient in the last moments of life, and thus, leaving them to die alone, clashes with the ethical responsibility to alleviate suffering [36].

In contrast with other studies conducted before the pandemic, the perception of administering futile treatments was remarkably low in our general sample. This could be related to two factors: on the one hand, to the therapeutic overexertion aimed at reversing serious situations caused by the Covid-19, such as the lack of oxygenation and the high mortality in critical care patients; and on the other hand, to the fact that treatment-limitation strategies implemented before admitting patients to ICUs were necessary and formed part of the decision-making process, due to the high demand for health care and the limitation of resources [33,37,38].

Another source of ethical conflict was related to the fact of not having sufficient personal protective equipment or enough human and material resources to take care of patients. As shown from the results for ECS-Co19 item 4, nurses had a moral dilemma between the obligation to care for patients and the need to ensure their own personal safety, as also reported in other studies [14,15,35].

Two variables increased the likelihood of the emergence of ethical conflict and psychological distress: being female and working in Covid-19 ICUs. The latter can be related to the fact that, during the pandemic, ICUs were overwhelmed by an increased number of patients in a short period of time, thus increasing the risk of having not enough human and material resources to guarantee the standard level of care [34,39]. With regard to the psychological impact of the pandemic, the survey indicated that 19.9% of the nurses reported cut-off scores for an initial post-traumatic stress syndrome symptomatology. The same result was also reported by Schroeder et al. (2020), who reported nurses’ anxiety levels, especially at the beginning of the pandemic [26].

Furthermore, nurses who worked in Covid-19 wards showed higher scores in each dimension of the IES-R, thus highlighting an increased level of psychological distress in this population. This result was confirmed by other studies on previous pandemics [5], as well as in the Covid-19 pandemic [40]. Moreover, nurses who were directly involved in the care of patients with Covid-19 reported significant work-psychological stress and burnout [41,42].

Based on the findings of this survey, the importance of monitoring the psychological well-being of nurses in order to prevent burnout syndrome or professional abandonment also clearly emerges [35,40,41]. This is even more true because, at the time of writing, we are in the middle of a third wave that may lead to what has been recently defined as pandemic fatigue [43].

Although up-to-date studies indicate the perception of a higher risk or fear of infection to be an important stress factor among health professionals [24], the data showing a moderate perception of risk emerging from the present study could be attributed to the emergency management strategies that the institution quickly implemented in order to deal with needs and deficiencies as they arose. For example, the fact of reorganising the hospitals and dedicating two of them solely to the care of Covid-19 patients had many positive outcomes: a better management of the emergency, which led to less discomfort for both Covid-19 ward nurses, who were able to focus all their attention on this type of situation, and for those who were not working in Covid-19 wards; an acceleration of the purchase and distribution of personal protection equipment; the possibility of offering temporary living-in accommodation in order to avoid contaminating family members; and the opportunity to keep professionals up-to-date, with daily communications on both the progress of the pandemic and the behavioural guidelines to be adopted. The importance of these aspects, and their protective role in terms of ethical conflict and psychological distress [5,44], is well covered in literature, which reports that resilience allows nurses to be flexible and adapt in a healthy manner to—or recover from—ethical conflicts while minimising their own suffering and preserving their integrity [45,46,47]. Furthermore, the high level of commitment and dedication of nurses to their job was confirmed by the professionals’ low level of intention to abandon their profession. This result could be explained by the higher level of resilience that was found in our sample: in fact, other research has demonstrated that resilience is negatively correlated with the intention of intensive care unit nurses to leave their profession [48,49].

This survey clearly indicates that some variables, such as work expertise and a higher level of resilience, seem to have a protective role against ethical conflict during the pandemic. This information is essential for the planning and management of future strategies focused on prevention and the preparation of nurses, both vis-à-vis their patients and themselves. As a consequence, being better prepared for future pandemics may imply the selection of a more experienced nursing team, because experience was found to be a protective factor from the point of view of ethical conflict.

### Limitations of the Study

This study presents several limitations. Despite 548 being a good sample size, it represents a 27.4% response rate of public hospital nurses in the Italian-speaking region of Switzerland. The large number of surveys in which nurses were asked to participate during the pandemic could have caused the low response rate. In fact, many other surveys were being conducted during the same period at the Ente Ospedaliero Cantonale. Furthermore, the online method of data collection may have influenced the response rate, as seen in other studies. The limited response rate, due to the fact a large proportion of nurses did not participate in the survey, may have influenced the results and, therefore, some findings may be biased. Nevertheless, the response rate was quantitatively comparable to other surveys in a similar setting.

Another limitation is related to the fact that this is a cross-sectional study and, therefore, the relationship between variables should be taken into careful consideration. In this respect, it could be interesting to repeat the survey to understand whether ethical conflict, psychological impact, and resilience changed during the pandemic: in the context in question, the management of the second and third waves of the pandemic was very different from the first one, from both a clinical and an organisational point of view.

## 7. Conclusions

The Covid-19 pandemic has had a significant impact on the level of ethical conflict, which was significantly correlated with psychological distress in our sample.

Significant differences emerged in levels of ethical conflict and psychological distress between nurses who worked in Covid-19 and non-Covid-19 wards. In particular, being female and working in Covid-19 ICUs increased the likelihood of suffering moral and psychological consequences. Within this context, the End-of-Life care and the related ethical responsibilities were found to be the main sources of conflict, despite the low levels of therapeutic futility perceived during this pandemic. This may have worsened the nurses’ well-being and increased the risk of burnout and work fatigue. As the situation is still critical and, at the time of writing, we are in the middle of a third wave of the pandemic, it is important to find strategies that protect nurses and other health professionals from ethical conflict and psychological distress as soon as possible.

## Figures and Tables

**Table 1:** Characteristics of the study participants.

Variables	All Nurses (N = 548)		Non-Covid-19 Wards (N = 273)		Covid-19 Wards (N = 275)		*p*-Value
Age, years	40	±10	40.8	±10.2	39.7	±10.1	0.8
Female	419	(76.5)	230	(84.2)	189	(76.5)	<0.001
Years of experience as nurse	N	%	N	%	N	%	
0 to 5	89	(16.2)	37	(13.2)	52	(18.9)	0.245
6 to 10	122	(22.3)	64	(23.4)	58	(21.1)	
11 to 20	137	(25)	65	(23.8)	76	(26.2)	
over 20	200	(36.5)	107	(39.2)	93	(33.8)	
Live alone	144	(26.3)	71	(26)	73	(26.5)	0.886
Live with frail people (i.e., children or older/ill people)	257	(46.9)	136	(49.9)	121	(44)	0.144
Post-basic education	317	(57.8)	148	(54.2)	169	(61.5)	0.086
Work-time percentage	N	%	N	%	N	%	
lower than 60%	34	(6.2)	25	(9.9)	9	(3.3)	<0.001
60 to 90%	213	(38.9)	130	(47.6)	83	(30.2)	
over 90%	301	(54.9)	118	(43.2)	183	(66.5)	
Relocation to another ward during the pandemic	167	(30.5)	37	(13.6)	130	(47.3)	<0.001
ICU during Covid-19	103	(18.8)	33	(12.1)	70	(25.5)	<0.001
Desire to change profession before the pandemic (yes/no)	147	(26.8)	76	(27.6)	71	(26.0)	0.67
Desire to change profession during the pandemic (yes/no)	119	(21.7)	66	(24)	53	(19.4)	0.19
Perception of the risk of being infected during the pandemic, (score 0 = low risk to 10 = high risk) (Median (25th, 75th percentiles))	5	[4–8]	6	[4–8]	4	[3–7]	<0.001
Perception of one’s own health status *(SF12 first question: How do you rate your health status on a scale from 1 = poor to 5 = excellent)* (Median [25th, 75th percentiles])	4	[2–4]	4	[2–4]	4	[2–4]	0.008

ICU: Intensive Care Unit; Covid-19: Coronavirus disease 2019; SF12: 12-Item Short Form Health Survey. Data are presented as counts (%) for categorical variables and mean (±standard deviation) for continuous variables, unless otherwise stated.

**Table 2:** Comparison of the ethical conflict score between Covid-19 and non-Covid-19 wards.

Ethical Conflict Scale Covid-19 (ECS-Co19) Items		All Nurses		Non-Covid-19 Wards		Covid-19 Wards		*p*-Value ^#^
		N = 548		N = 273		N = 275		
1	I perceived more ethical conflicts during the Covid-19 crisis than before.	9	[4.0–12.0] ^ç^	8.0	[4.0–9.0]	9.0	[4.0–12.0]	<0.001
2	I suffered for not being able to adequately care for patients due to lack of material resources, space or equipment.	6	[4.0–12.0]	4.0	[2.5–9.0]	9.0	[4.0–16.0]	<0.001
3	I suffered for not being able to adequately care for patients due to a lack of staff.	4	[1.0–9.0]	4.0	[1.0–8.5]	6.0	[2.0–15.0]	<0.001
4	The moral obligation to care for patients outweighed the need to ensure my personal safety.	6	[3.0–12.0]	4.0	[2.0–9.0]	8.0	[4.0–12.0]	<0.001
5	During the current health crisis, respect for patient autonomy has been undermined.	6	[2.0–9.0]	4.0	[1.0–9.0]	6.0	[4.0–10.0]	<0.001
6	I suffered for the fact that End-of-Life care for patients could not be guaranteed as before.	6	[2.0–16.0]	4.0	[1.0–9.0]	10.0	[4.0–16.0]	<0.001
7	I suffered for seeing patients dying alone.	9	[4.0–16.0]	5.0	[1.0–12.0]	15.0	[8.0–20.0]	<0.001
8	I provided care and/or treatment that I did not consider necessary.	2	[1.0–5.0]	1.0	[1.0–4.0]	3.0	[1.0–6.0]	0.012
9	Directives were agreed to limit life support treatment in patients in certain situations.	4	[1.0–8.0]	2.0	[1.0–4.0]	6.0	[2.0–12.0]	<0.001
10	I suffered for communicating bad news to family members by telephone and/or video calls.	4	[1.0–6.0]	4.0	[1.0–5.0]	4.0	[1.0–9.0]	0.003
11	I suffered for having to care for a patient for whom I felt I did not have the right skills.	4	[1.0–6.0]	1.0	[1.0–4.0]	4.0	[1.0–9.0]	<0.001
12	I suffered for working with colleagues who I felt did not have the right skills.	4	[1.0–6.0]	3.0	[1.0–5.0]	4.0	[1.0–9.0]	<0.001
13	I suffered for having to prioritise between who was more likely to benefit from treatment and who was less likely to.	3	[1.0–6.0]	1.0	[1.0–4.0]	4.0	[1.0–9.0]	<0.001
14	I suffered because I was not always able to provide treatments to alleviate pain and suffering when needed.	4	[1.0–8.0]	3.0	[1.0–5.0]	5.0	[1.0–9.0]	<0.001
15	I suffered because I could see that some negative outcomes for the patient depended on the quality of nursing care.	4	[1.0–5.0]	1.0	[1.0–5.0]	4.0	[1.0–6.0]	0.005
16	I suffered for having to choose patients who got life-saving treatment and those who did not.	1	[1.0–5.0]	1.0	[1.0–5.0]	2.0	[1.0–8.0]	0.002
17	I suffered for having to care for colleagues who had contracted Covid-19.	1	[1.0–4.0]	1.0	[1.0–4.0]	1.0	[1.0–5.0]	0.988
	Total score	111.5	[76.0–152.0]	91.0	[68.5–125.0]	133.0	[91.0–179.0]	<0.001

Covid-19: Coronavirus disease 2019. ^ç^ Data are presented as median, 25th and 75th percentiles. ^#^ Mann–Whitney U test.

**Table 3:** Comparison of the IES-R score and its subscales between Covid-19 and non-Covid-19 wards.

Impact of Event Scale (IES)-Revised Scoring Sum of the Item Values	All Nurses (548)		Non-Covid-19 Wards (N = 273)		Covid-19 Wards (N = 275)		*p*-Value ^#^
IES Subscale Avoidance	5	[2–8.75] ^ç^	4	[1–8]	5	[2–9]	0.029
IES Subscale Intrusion	6	[3–11]	5	[2–10]	7	[3–14]	<0.001
IES Subscale hyper-arousal	4	[1–7]	3	[1–6]	4	[1–8]	0.301
IES Impact of Event Scale (0–88) (Total score)	15	[7–26]	12	[6–23.5]	17	[7–29]	0.005
IES-R > 30 N (%)	109 (19.9)		41 (15.0)		62 (24.7)		<0.001

IES-R: Impact Event Scale-Revised. ^ç^ Data are presented as median, 25th and 75th percentiles. ^#^ Mann–Whitney U test.

**Table 4:** Brief Resilience Scale (By Smith et al., 2008).

Variables	All Nurses (N = 548)		Covid-19 Wards (N = 275)		Non-Covid-19 Wards (N = 273)		*p*-Value ^#^
Brief Resilience Scale—usual resilience, (score 0 to 5)	3	[2.8–3.2] ^ç^	3	[2.8–3]	3	[2.8–3.2]	0.23
Brief Resilience Scale—during the pandemic, (score 0 to 5)	3	[2.8–3.0]	3	[2.8–3]	3	[2.8–3]	0.57

Covid-19: Coronavirus disease 2019; ^ç^ data are presented as median, 25th and 75th percentiles. ^#^ Mann–Whitney U test.

**Table 5:** Predictive variables of high ethical conflict and high levels of psychological distress.

Variables	High Ethical Conflict		IES-R > 30 Points	
	OR (95% CI)	*p*-Value	OR (95% CI)	*P*-Value
Female	2.62 (1.56–4.39)	<0.001	2.54 (1.38–4.66)	0.003
Years of experience as nurse	0.69 (0.56–0.86)	0.001	1.08 (0.86–1.36)	0.468
Post-basic education	1.25 (0.77–2.02)	0.352	0.95 (0.57–1.59)	0.865
Full-time job	0.92 (0.59–1.45)	0.743	1.33 (0.82–2.18)	0.242
Desire to change profession before the pandemic	1.21 (0.78–1.89)	0.378	1.15 (0.71–1.86)	0.555
Brief Resilience Scale (usual resilience)	0.50 (0.26–0.94)	0.033	0.81 (0.42–1.59)	0.558
Hospital wards				
General ward	(reference)	NA	(reference)	NA
Covid-19 ward	5.85 (3.56–9.61)	<0.001	2.16 (1.29–3.60)	0.003
ICU	3.18 (1.41–7.16)	<0.005	1.04 (0.39–2.74)	0.929
Covid-19 ICU	7.18 (3.96–13.01)	<0.001	1.75 (0.93–3.31)	0.083
	HL-test *p* = 0.124 AUC = 75 (CI 95% 71–80)		HL-test *p* = 0.458 AUC = 64 (CI 95% 58–69)	

Covid-19: Coronavirus disease 2019; IES-R: Impact Event Scale-Revised; ICU: Intensive Care Unit; OR: odds ratio; CI: confidence interval; HL-test: Hosmer–Lemeshow test; AUC: Area Under the Curve; NA: Not Applicable.

## Data Availability

The data presented in this study are available on request from the corresponding author. The data are not publicity available due to privacy and institutional restrictions.

## Supplementary Materials

The following are available online at https://www.mdpi.com/article/10.3390/ijerph182212012/s1, S1: The final version of the ethical conflict covid-19 (ECS Co-19) scale and explorative factor analysis, Cronbach alpha and intra-class correlation. S2: The predictive ability of the logistic regression model to discriminate between nurses who experienced any consequences and those who did not, estimated by the Receiver Operating Characteristic (ROC).
