# Supplementary material for: Ethical Conflict and Its Psychological Correlates among Hospital Nurses in the Pandemic: A Cross-Sectional Study within Swiss COVID-19 and Non-COVID-19 Wards
Source: Int J Environ Res Public Health. 2021 Nov 16;18(22):12012. doi: 10.3390/ijerph182212012 (PMC8618535; doi:10.3390/ijerph182212012)

SUPPLEMENTARY MATERIALS 1 (S1): FINAL VERSION OF THE ETHICAL CONFLICT SCALE COVID-19 (ECS CO-19) AND EXPLORATIVE FACTOR ANALYSIS, CRONBACH ALPHA AND ITRA-CLASS CORRELATION

Ethical Conflict Scale Covid-19 (ECS Co-19) scale (final version)

	**Area**	**Ethical Conflict Scale Covid-19 (ECS Co-19) Items**
1	Resources	I perceived more ethical conflicts during the Covid-19 crisis than before.
2	Resources	I suffered for not being able to adequately care for patients due to lack of material resources, space or equipment.
3	Resources	I suffered for not being able to adequately care for patients due to a lack of staff.
4	Protection	The moral obligation to care for patients outweighed the need to ensure my personal safety.
5	Protection	During the current health crisis, respect for patient autonomy has been undermined.
6	EOL	I suffered for the fact that End-of-Life care for patients could not be guaranteed as before.
7	EOL	I suffered for seeing patients dying alone.
8	EOL	I provided care and/or treatment that I did not consider necessary.
9	EOL	Directives were agreed to limit life support treatment in patients in certain situations.
10	ICT	I suffered for communicating bad news to family members by telephone and/or video calls.
11	Resources	I suffered for having to care for a patient for whom I felt I did not have the right skills.
12	Resources	I suffered for working with colleagues who I felt did not have the right skills.
13	Decisions	I suffered for having to prioritise between who was more likely to benefit from treatment and who was less likely to.
14	EOL	I suffered because I was not always able to provide treatments to alleviate pain and suffering when needed.
15	Decisions	I suffered because I could see that some negative outcomes for the patient depended on the quality of nursing care.
16	Decisions	I suffered for having to choose patients who got life-saving treatment and those who did not
17	Decisions	I suffered for having to care for colleagues who had contracted Covid-19.

Resources: Availability of material resources and professionals for care; **Protection:** Adequate protection for healthcare workers to prevent Covid-19 infection; **Decisions:** Decisions making process during Covid-19 pandemic; **EOL:** End-of-life care and withholding and withdrawal treatment; **ICT:** Information and communication technologies to be in touch with relatives

Deleted item after EFA procedure:

- The use of tablets/cell phones to allow patients to communicate with their families caused me conflict regarding the protection of privacy.

The ECS Co-19 scale is protected by copyright. The permission to use or translate the ECS Co-19 scale must be asked to the authors of the original tool (LB, AFP, MV and SB).

Authors contact:

LB: loris.bonetti@eoc.ch; AFP: annafalco@ub.edu; MV: michele.villa@eoc.ch; SR: serena.barello@unicatt.it

Explorative faxtor analys 18 items

KMO and Bartlett's Test

Kaiser-Meyer-Olkin Measure of Sampling Adequacy.		,906
Bartlett's Test of Sphericity	Approx. Chi-Square	3209,075
	df	153
	Sig.	,000

Communalities

	Initial	Extraction
Item 1 I perceived more ethical conflicts during the Covid-19 crisis than before.	,353	,389
Item 2 I suffered for not being able to adequately care for patients due to lack of material resources, space or equipment.	,487	,586
Item 3 I suffered for not being able to adequately care for patients due to a lack of staff.	,507	,595
Item 4 The moral obligation to care for patients outweighed the need to ensure my personal safety.	,251	,269
Item 5 During the current health crisis, respect for patient autonomy has been undermined.	,245	,227
Item 6 I suffered for the fact that End-of-Life care for patients could not be guaranteed as before.	,501	,626
Item 7 The use of tablets/cell phones to allow patients to communicate with their families caused me conflict regarding the protection of privacy.	,104	,136
Item 8 I suffered for seeing patients dying alone.	,463	,597
Item 9 I provided care and/or treatment that I did not consider necessary.	,205	,300
Item 10 Directives were agreed to limit life support treatment in patients in certain situations.	,439	,557
Item 11 I suffered for communicating bad news to family members by telephone and/or video calls.	,297	,417
Item 12 I suffered for having to care for a patient for whom I felt I did not have the right skills.	,262	,251
Item 13 I suffered for working with colleagues who I felt did not have the right skills.	,312	,390
Item 14 I suffered for having to prioritise between who was more likely to benefit from treatment and who was less likely to.	,562	,622
Item 15 I suffered because I was not always able to provide treatments to alleviate pain and suffering when needed.	,436	,469
Item 16 I suffered because I could see that some negative outcomes for the patient depended on the quality of nursing care.	,382	,464
Item 17 I suffered for having to choose patients who got life-saving treatment and those who did not	,548	,691
Item 18 I suffered for having to care for colleagues who had contracted Covid-19.	,237	,342

Extraction Method: Principal Axis Factoring.

Total Variance Explained

Factor	Initial Eigenvalues			Extraction Sums of Squared Loadings		
	Total	% of Variance	Cumulative %	Total	% of Variance	Cumulative %
1	6,266	34,810	34,810	5,760	32,003	32,003
2	1,230	6,834	41,643	,746	4,142	36,145
3	1,091	6,060	47,704	,565	3,136	39,281
4	1,066	5,923	53,627	,461	2,564	41,845
5	1,017	5,650	59,277	,395	2,193	44,038
6	,876	4,868	64,145			
7	,780	4,331	68,476			
8	,757	4,207	72,683			
9	,692	3,844	76,528			
10	,625	3,474	80,001			
11	,618	3,431	83,432			
12	,596	3,308	86,741			
13	,515	2,858	89,599			
14	,458	2,546	92,145			
15	,407	2,260	94,405			
16	,397	2,205	96,610			
17	,337	1,873	98,484			
18	,273	1,516	100,000			

Extraction Method: Principal Axis Factoring.

PARALLEL ANALYSIS (18 items):

Principal Components

Specifications for this Run:

Ncases	548
Nvars	18
Ndatsets	100
Percent	95

Random Data Eigenvalues

Root	Means	Prcntyle
1,000000	1,326338	1,380324
2,000000	1,262877	1,307791
3,000000	1,216078	1,252344
4,000000	1,173996	1,199977
5,000000	1,137785	1,162322
6,000000	1,103961	1,126634
7,000000	1,069857	1,092835
8,000000	1,039436	1,062732
9,000000	1,005738	1,028928
10,000000	,977732	1,005337
11,000000	,946211	,969676
12,000000	,913901	,940409
13,000000	,886303	,913164
14,000000	,854782	,880500
15,000000	,826741	,848254
16,000000	,792680	,819395
17,000000	,754152	,789187
18,000000	,711433	,746117

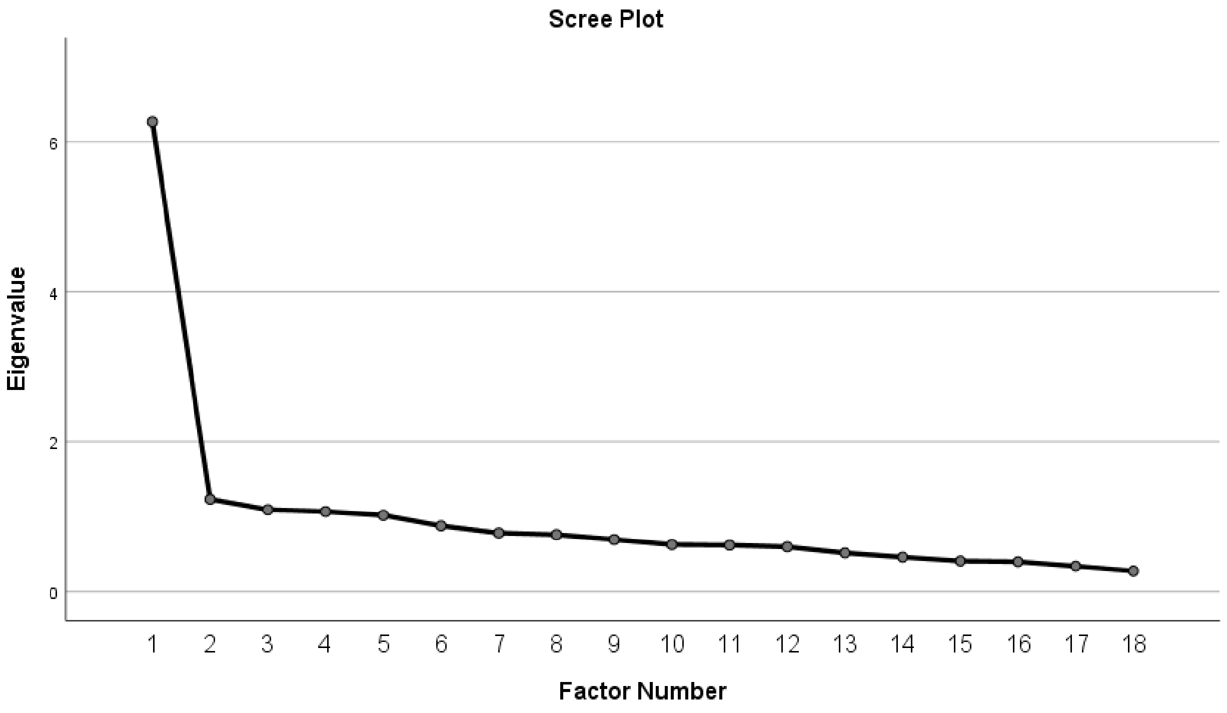

Factor Matrix[a]

	Factor				
	1	2	3	4	5
Item 14 I suffered for having to prioritise between who was more likely to benefit from treatment and who was less likely to.	,726	-,286	-,017	-,095	,067
Item 17 I suffered for having to choose patients who got life-saving treatment and those who did not	,681	-,456	,020	-,135	,011
Item 2 I suffered for not being able to adequately care for patients due to lack of material resources, space or equipment.	,671	,278	,090	-,003	,226
Item 6 I suffered for the fact that End-of-Life care for patients could not be guaranteed as before.	,668	,244	-,335	-,055	-,067
Item 3 I suffered for not being able to adequately care for patients due to a lack of staff.	,661	,243	,263	-,118	,125
Item 15 I suffered because I was not always able to provide treatments to alleviate pain and suffering when needed.	,649	-,009	-,025	,179	-,124
Item 8 I suffered for seeing patients dying alone.	,627	,144	-,416	-,006	-,098
Item 10 Directives were agreed to limit life support treatment in patients in certain situations.	,618	-,213	-,049	-,333	-,131
Item 1 I perceived more ethical conflicts during the Covid-19 crisis than before.	,593	,112	,022	-,122	,096
Item 16 I suffered because I could see that some negative outcomes for the patient depended on the quality of nursing care.	,572	-,162	,259	,136	-,156
Item 13 I suffered for working with colleagues who I felt did not have the right skills.	,516	,128	,285	-,006	-,162
Item 11 I suffered for communicating bad news to family members by telephone and/or video calls.	,495	-,159	-,122	,356	,068
Item 12 I suffered for having to care for a patient for whom I felt I did not have the right skills.	,476	,118	-,074	,056	-,049
Item 4 The moral obligation to care for patients outweighed the need to ensure my personal safety.	,469	,149	,099	-,059	,113
Item 5 During the current health crisis, respect for patient autonomy has been undermined.	,462	,113	,021	,024	,004
Item 18 I suffered for having to care for colleagues who had contracted Covid-19.	,429	-,249	-,062	,130	,274
Item 9 I provided care and/or treatment that I did not consider necessary.	,390	,051	,115	,177	-,317
Item 7 The use of tablets/cell phones to allow patients to communicate with their families caused me conflict regarding the protection of privacy.	,232	,006	,039	,242	,147

Extraction Method: Principal Axis Factoring.

a. 5 factors extracted. 9 iterations required.

Explorative factor analysis 17 items

KMO and Bartlett's Test

Kaiser-Meyer-Olkin Measure of Sampling Adequacy.		,907
Bartlett's Test of Sphericity	Approx. Chi-Square	3151,891
	df	136
	Sig.	,000

Communalities

	Initial	Extraction
Item 1 I perceived more ethical conflicts during the Covid-19 crisis than before.	,352	,390
Item 2 I suffered for not being able to adequately care for patients due to lack of material resources, space or equipment.	,483	,537
Item 3 I suffered for not being able to adequately care for patients due to a lack of staff.	,507	,604
Item 4 The moral obligation to care for patients outweighed the need to ensure my personal safety.	,251	,270
Item 5 During the current health crisis, respect for patient autonomy has been undermined.	,244	,227
Item 6 I suffered for the fact that End-of-Life care for patients could not be guaranteed as before.	,499	,613
Item 8 I suffered for seeing patients dying alone.	,461	,589
Item 9 I provided care and/or treatment that I did not consider necessary.	,199	,262
Item 10 Directives were agreed to limit life support treatment in patients in certain situations.	,434	,421
Item 11 I suffered for communicating bad news to family members by telephone and/or video calls.	,286	,287
Item 12 I suffered for having to care for a patient for whom I felt I did not have the right skills.	,261	,253
Item 13 I suffered for working with colleagues who I felt did not have the right skills.	,312	,370
Item 14 I suffered for having to prioritise between who was more likely to benefit from treatment and who was less likely to.	,562	,633
Item 15 I suffered because I was not always able to provide treatments to alleviate pain and suffering when needed.	,434	,491
Item 16 I suffered because I could see that some negative outcomes for the patient depended on the quality of nursing care.	,382	,480
Item 17 I suffered for having to choose patients who got life-saving treatment and those who did not	,548	,713
Item 18 I suffered for having to care for colleagues who had contracted Covid-19.	,225	,230

Extraction Method: Principal Axis Factoring.

Total Variance Explained

Factor	Initial Eigenvalues			Extraction Sums of Squared Loadings		
	Total	% of Variance	Cumulative %	Total	% of Variance	Cumulative %
1	6,209	36,523	36,523	5,689	33,464	33,464
2	1,227	7,215	43,738	,729	4,290	37,754
3	1,068	6,282	50,020	,553	3,251	41,005
4	1,022	6,011	56,032	,399	2,348	43,353
5	,896	5,268	61,300			
6	,864	5,084	66,384			
7	,758	4,456	70,840			
8	,692	4,072	74,912			
9	,632	3,718	78,630			
10	,624	3,669	82,300			
11	,600	3,529	85,829			
12	,529	3,111	88,940			
13	,458	2,697	91,637			
14	,411	2,417	94,053			
15	,400	2,353	96,406			
16	,338	1,988	98,394			
17	,273	1,606	100,000			

Extraction Method: Principal Axis Factoring.

PARALLEL ANALYSIS (17 items):

Principal Components

Specifications for this Run:

Ncases	548
Nvars	17
Ndatsets	100
Percent	95

Random Data Eigenvalues

Root	Means	Prcntyle
1,000000	1,319523	1,384962
2,000000	1,255549	1,291576
3,000000	1,204301	1,232476
4,000000	1,159350	1,190203
5,000000	1,124512	1,150474
6,000000	1,090338	1,114643
7,000000	1,056978	1,085017
8,000000	1,021404	1,041330
9,000000	,992514	1,019152
10,000000	,962094	,990139
11,000000	,929792	,957962
12,000000	,898259	,920154
13,000000	,864556	,891749
14,000000	,833471	,865261
15,000000	,801180	,833966
16,000000	,766090	,799345
17,000000	,720088	,761501

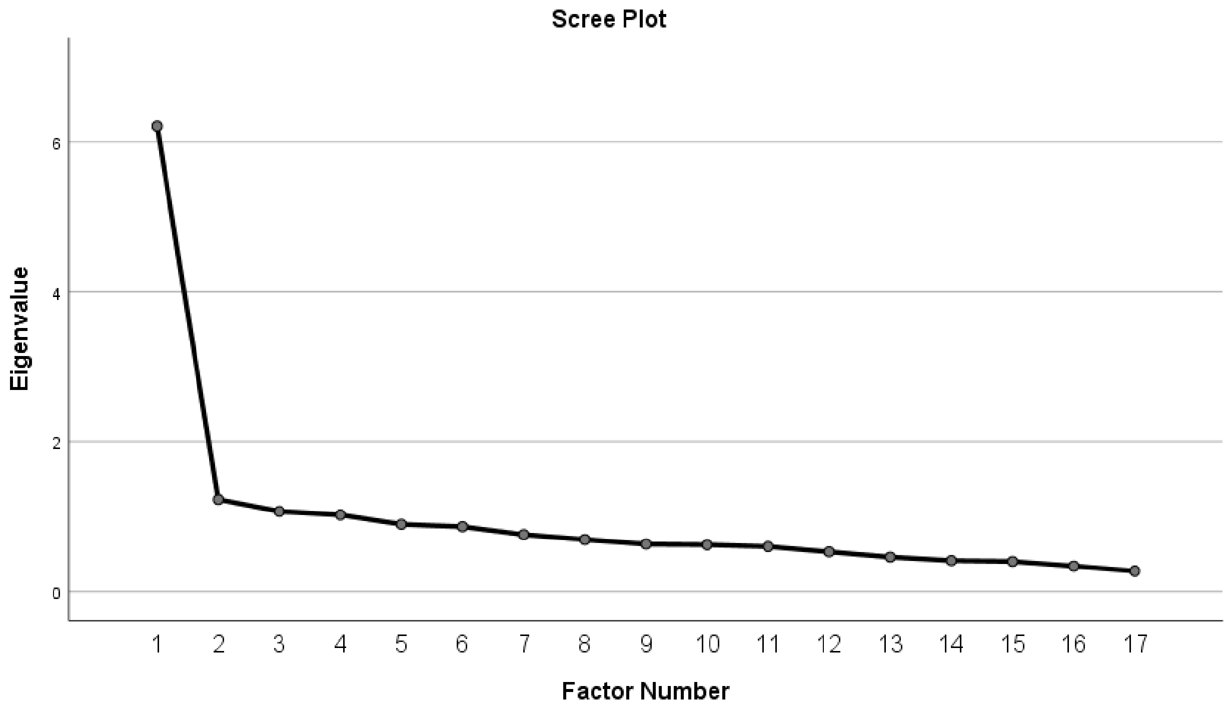
Scree Plot
Eigenvalue
0
2
4
6
1
2
3
4
5
6
7
8
9
10
11
12
13
14
15
16
17
Factor Number

Factor Matrix[a]				
	Factor			
	1	2	3	4
Item 14 I suffered for having to prioritise between who was more likely to benefit from treatment and who was less likely to.	,729	-,292	-,034	-,124
Item 17 I suffered for having to choose patients who got life-saving treatment and those who did not	,686	-,479	-,010	-,116
Item 6 I suffered for the fact that End-of-Life care for patients could not be guaranteed as before.	,668	,254	-,320	,001
Item 2 I suffered for not being able to adequately care for patients due to lack of material resources, space or equipment.	,664	,258	,096	-,141
Item 3 I suffered for not being able to adequately care for patients due to a lack of staff.	,664	,235	,285	-,165
Item 15 I suffered because I was not always able to provide treatments to alleviate pain and suffering when needed.	,654	-,012	-,031	,250
Item 8 I suffered for seeing patients dying alone.	,629	,163	-,403	,065
Item 10 Directives were agreed to limit life support treatment in patients in certain situations.	,609	-,182	-,046	-,123
Item 1 I perceived more ethical conflicts during the Covid-19 crisis than before.	,595	,111	,029	-,153
Item 16 I suffered because I could see that some negative outcomes for the patient depended on the quality of nursing care.	,575	-,180	,257	,225
Item 13 I suffered for working with colleagues who I felt did not have the right skills.	,515	,110	,283	,108
Item 11 I suffered for communicating bad news to family members by telephone and/or video calls.	,481	-,131	-,114	,162
Item 12 I suffered for having to care for a patient for whom I felt I did not have the right skills.	,476	,118	-,075	,079
Item 4 The moral obligation to care for patients outweighed the need to ensure my personal safety.	,471	,144	,108	-,127
Item 5 During the current health crisis, respect for patient autonomy has been undermined.	,462	,114	,029	,005
Item 18 I suffered for having to care for colleagues who had contracted Covid-19.	,417	-,214	-,069	-,074
Item 9 I provided care and/or treatment that I did not consider necessary.	,386`	,039	,102	,318

Extraction Method: Principal Axis Factoring.

a. 4 factors extracted. 12 iterations required.

Scale: Cronbach Alpha Ethical Conflict Covid-19 Scale

Reliability Statistics

Cronbach's Alpha	N of Items
,887	17

Item-Total Statistics

	Scale Mean if Item Deleted	Scale Variance if Item Deleted	Corrected Item-Total Correlation	Cronbach's Alpha if Item Deleted
Item 1 I perceived more ethical conflicts during the Covid-19 crisis than before.	95,65	2938,353	,567	,879
Item 2 I suffered for not being able to adequately care for patients due to lack of material resources, space or equipment.	95,88	2835,266	,631	,876
Item 3 I suffered for not being able to adequately care for patients due to a lack of staff.	97,02	2823,420	,613	,877
Item 4 The moral obligation to care for patients outweighed the need to ensure my personal safety.	96,63	2985,927	,451	,883
Item 5 During the current health crisis, respect for patient autonomy has been undermined.	97,36	3003,346	,447	,883
Item 6 I suffered for the fact that End-of-Life care for patients could not be guaranteed as before.	95,65	2772,611	,630	,876
Item 8 I suffered for seeing patients dying alone.	93,17	2746,017	,584	,880
Item 9 I provided care and/or treatment that I did not consider necessary.	100,41	3134,944	,360	,886
Item 10 Directives were agreed to limit life support treatment in patients in certain situations.	99,01	2963,501	,564	,879
Item 11 I suffered for communicating bad news to family members by telephone and/or video calls.	99,35	3034,721	,448	,883
Item 12 I suffered for having to care for a patient for whom I felt I did not have the right skills.	99,49	3025,621	,455	,883
Item 13 I suffered for working with colleagues who I felt did not have the right skills.	99,18	2995,852	,481	,882
Item 14 I suffered for having to prioritise between who was more likely to benefit from treatment and who was less likely to.	99,55	2908,123	,665	,876
Item 15 I suffered because I was not always able to provide treatments to alleviate pain and suffering when needed.	99,21	2956,170	,613	,878
Item 16 I suffered because I could see that some negative outcomes for the patient depended on the quality of nursing care.	99,97	3037,244	,523	,881
Item 17 I suffered for having to choose patients who got life-saving treatment and those who did not	100,17	2967,977	,601	,878
Item 18 I suffered for having to care for colleagues who had contracted Covid-19.	101,03	3140,648	,382	,885

Item	Intra-Class Correlation (ICC)*	CI 95%		*p value*
**1**	.945	.727	.989	.001
**2**	.964	.813	.993	<.001
**3**	.994	.972	.999	<.001
**4**	.945	.749	.989	.001
**5**	.981	.910	.996	<.001
**6**	.983	.918	.997	<.001
**7**	.998	.972	.999	<.001
**8**	.975	.884	.995	<.001
**9**	.975	.884	.995	.001
**10**	.807	- .080	.962	.03
**11**	.750	- .028	.948	.032
**12**	.991	.956	.998	<.001
**13**	.976	.891	.995	<.001
**14**	.995	.977	.999	<.001
**15**	.883	.480	.976	.005
**16**	.965	.841	.993	.001
**17**	.972	.862	.994	<.001
**Total score**	.995	.895	.999	<.001

*We used absolute agreement ICC

SUPPLEMENTARY MATERIALS 2 (S2): predictive ability of the logistic regression model to discriminate between nurses who experienced any consequences and those who did not, estimated by the Receiver Operating Characteristic (ROC)

Figure S1: ROC Ethical Conflict Scale Covid-19 (ECS-Co19) (Area Under Curve (AUC)=75 (CI 95% 71-80)).

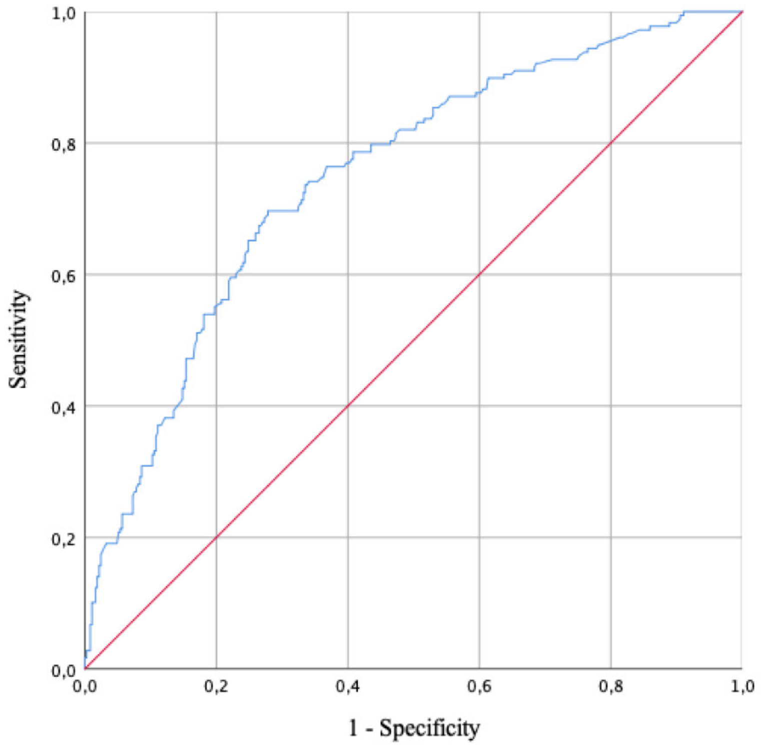

Figure S2: ROC Impact of Event Scale Revised (IES-R) (Area Under Curve(AUC)=64 (CI 95% 58-69)).

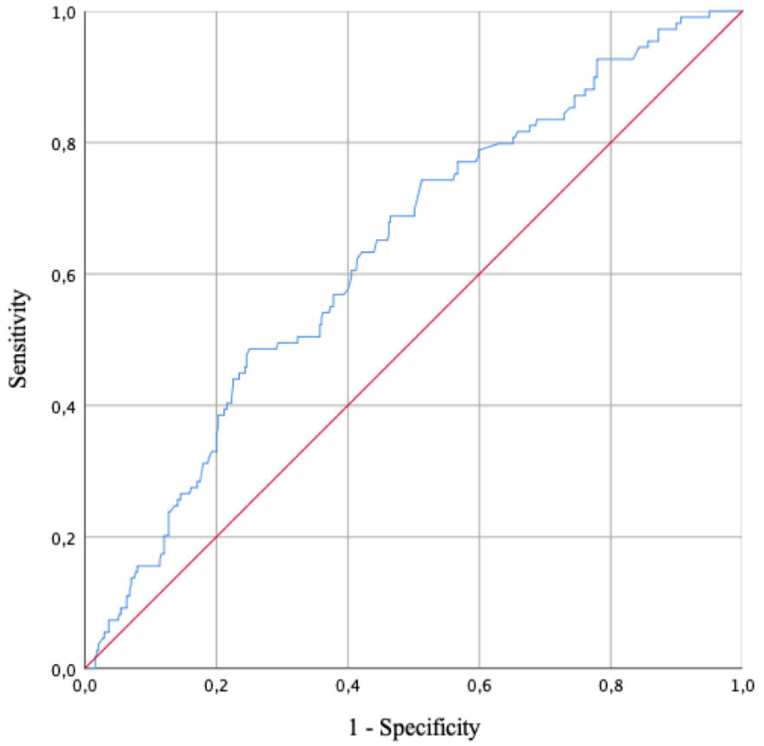

Supplement: Supplementary file 1 [file ijerph-18-12012-s001.zip › ijerph-1426174-supplementary.pdf]
